# Comparison of sound touch elastography and quantification for assessing the renal pathologic changes in patients with proteinuria

**DOI:** 10.1186/s13244-023-01476-9

**Published:** 2023-08-04

**Authors:** Zhengmin Ruan, Zhiying Xiao, Xue Shi, Yu Liang, Liang Hou, Tao Wu, Mei Wu

**Affiliations:** 1 Department of Ultrasound, The Second Hospital of Shandong University, No 247, Beiyuan Street, Jinan, 250033 Shandong China; 2 Department of Urology, The Second Hospital of Shandong University, Jinan, Shandong China; 3 Department of Nephrology, The Second Hospital of Shandong University, Jinan, Shandong China

**Keywords:** Sound touch elastography, Sound touch quantification, Shear wave, Ultrasonography, Chronic kidney disease

## Abstract

**Objective:**

Sound touch elastography (STE) and sound touch quantification (STQ) are novel imaging methods to evaluate tissue stiffness. This study aims to investigate renal stiffness in patients with chronic kidney disease (CKD) by STE and STQ, using renal biopsy as ‘gold standard’.

**Methods:**

Between 2019 January and 2022 June, 60 patients who underwent renal biopsy for proteinuria (cases) and 45 healthy volunteers (controls) at our hospital were included in this study. The maximum and mean elastic modulus (Emax, Emean) of region of interest in right kidney were measured by STE and STQ techniques. Biochemical profiles and renal biopsy findings were recorded.

**Results:**

Both Emax and Emean measured by STE were significantly different between cases and controls. ROC analysis of STE measurements revealed using a cutoff of 13.53 kPa for Emax and 10.16 kPa for Emean, the area under the curve (AUC) to distinguish nephropathy from healthy was 0.718 and 0.744. Analysis of ROC for STQ measurements showed that using a cutoff value of 15.87 kPa for Emax and 9.95 kPa for Emean, the AUC for the nephropathy was 0.612 and 0.569. Emax and Emean values were significantly different among CKD patients with mild, moderate and severe pathological stage. The Emax value for STE was positively related to Scr, β2-MG (*r* = 0.257, 0.292, *p* < 0.05).

**Conclusion:**

Both STE and STQ are non-invasive, feasible methods to quantitatively evaluate renal stiffness. STE is more effective than STQ in the diagnosis of CKD patients with proteinuria.

**Critical relevance statement:**

Sound touch elastography is more effective than sound touch quantification in the diagnosis of chronic kidney disease patients with proteinuria.

**Key points:**

• Emax and Emean measured by STE were different between cases and controls.

• Emax and Emean were different among CKD patients with different pathological stages.

• The Emax value for STE was positively related to serum creatinine, β2-microglobulin

**Graphical Abstract:**

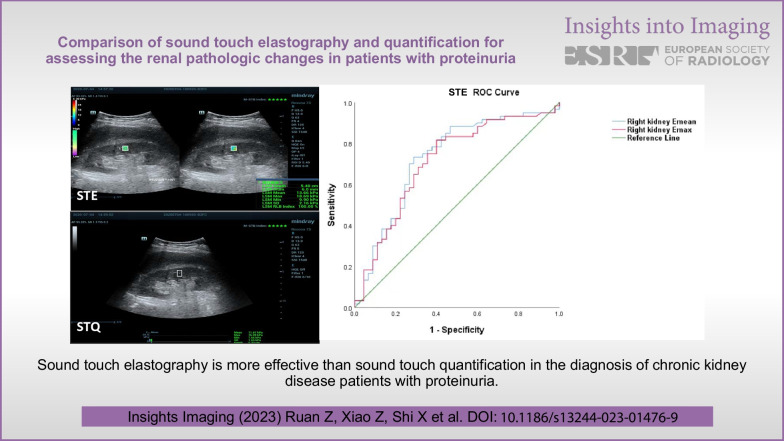

## Introduction

Chronic kidney disease (CKD) is defined as structural or functional abnormalities of the kidney for ≥ 3 months [1]. It is a global health problem, affecting 10.6–13.4% adults [2]. The clinical presentations can vary from asymptomatic or non-specific symptoms such as nausea, fatigue, to edema, proteinuria or even death. Proteinuria can serve as an indicator of early renal disease, and has been used along with the estimated glomerular filtration rate (eGFR) in the classification of CKD. Proteins in the urine can cause glomerulosclerosis and lead to tubulointerstitium disease [3].

The progression of CKD is characterized histologically by glomerulosclerosis, tubulointerstitial fibrosis, and vascular sclerosis [4, 5]. Renal biopsy is currently the gold standard for assessing renal fibrosis and CKD diagnosis. However, even it is a minimally invasive procedure, there are risks of complications with vascular complications. It is believed the risk of bleeding during renal biopsy increased with the severity of renal failure [6]. Therefore, a non-invasive method instead of repeated biopsy is essential in active monitoring of CKD.

Ultrasonography (US) is a safe, affordable and non-invasive procedure to examine the kidneys. Renal length, cortical thickness and echogenicity measured by ultrasound could help to assess renal function impairment in CKD patients [7, 8]. However, US is a highly examiner-dependent, non-quantitative imaging technique. Abnormal US findings usually represent advanced rather than early stage of renal dysfunction [9]. Previous studies have reported that routine US had limited diagnostic utility in CKD classification [10–12].

Ultrasonic shear wave elastography (SWE) is a non-invasive ultrasound elastography technique that uses shear waves to quantitatively measure tissue stiffness and it has been used to determine the stiffness of liver, kidney, thyroid and muscles [13–16]. A recent study reported that SWE was an effective way to evaluate the changes in the stiffness and elasticity of the renal parenchyma in diabetic nephropathy [17]. However, the relationship between renal fibrosis and stiffness still remains uncertain in various studies [18, 19].

Sound touch elastography (STE) and sound touch quantification (STQ) are two modified shear wave elastography methods to evaluate tissue stiffness [13]. STE/STQ use acoustic radiation force impulse (ARFI) elastography techniques to generate shear waves, then tracks the propagation of the shear waves and continuously detects and records the displacement of tissue in the region of interest (ROI) by ultra-wide beam tracking imaging technology. STQ is a point SWE method that uses ARFI to aim for one small ROI to generate a quantitative reading. A larger shear wave velocity (SWV) means a higher stiffness of the tissue. It usually indicates the average rather than maximum elastic modulus of the ROI [20]. In contrast, STE is a bi-dimensional SWE (2D-SWE) method that uses multiple ARFI to target an extended ROI to display a real-time colored stiffness map [21]. It can calculate the SWV and derives the corresponding and maximum elastic modulus [20]. The STE/STQ technology ensures that the shear wave can be recorded more rapidly, accurately, and completely than conventional SWE. There are few studies evaluating renal stiffness using STE or STQ.

The present study aimed to quantitatively analyze renal fibrosis in those patients with unexplained proteinuria using STE and STQ.

## Patients and methods

### Patient population

**Fig. 1:**
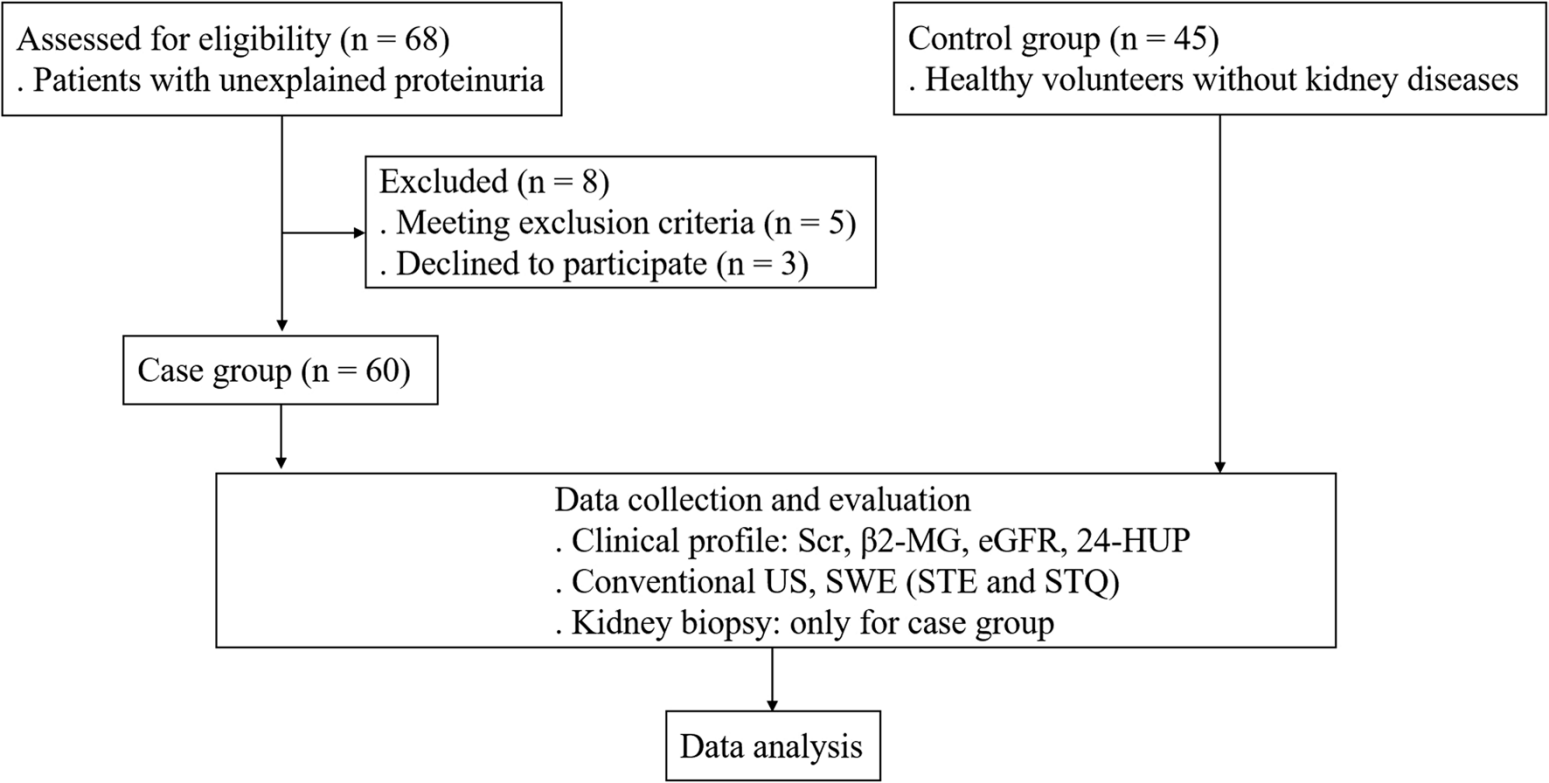
Flow chart of the study. *Scr* serum creatinine, *β2-MG* β2-microglobulin, *eGFR* estimated glomerular filtration rate, *24-HUP* 24-h urine protein, *US* ultrasonography, *SWE* shear wave elastography, *STE* sound touch elastography, *STQ* sound touch quantification

A prospective study was conducted on consecutive patients admitted to the Department of Nephrology in our hospital between 2019 January and 2022 June. Patients with unexplained proteinuria who underwent kidney biopsy were recruited. Those patients with proteinuria who had preexisting hypertension, diabetes, nephrotic syndrome or immunologic nephritis also met inclusion criteria. Patients were excluded if they were not able to attend kidney biopsy or kidney ultrasound elastography; if infection, stone or tumor induced proteinuria; in case the patients had renal replacement therapy, kidney transplant, hydronephrosis, renal calculus or tumor. One hundred and five subjects were finally enrolled and analyzed, which included 60 patients with proteinuria (“cases”) and 45 healthy volunteers (“controls”) from the Physical Examination Center in our hospital. Those individuals with abnormal urinalysis, kidney US or renal function were not eligible in the control group (Fig. 1).

This study was approved by the ethics committee of our hospital and was performed in accordance with the ethical guidelines of the Helsinki Declaration and its later amendments. Written informed consent was obtained from all participants.

Demographic data including age, sex, height, weight and body mass index (BMI) were collected. The clinical profile including kidney biopsy, serum creatinine (Scr), β2-microglobulin (β2-MG), glomerular filtration rate (eGFR) and 24-h urine protein (24-HUP) were recorded.

### Conventional renal ultrasonography and shear wave elastography

**Fig. 2:**
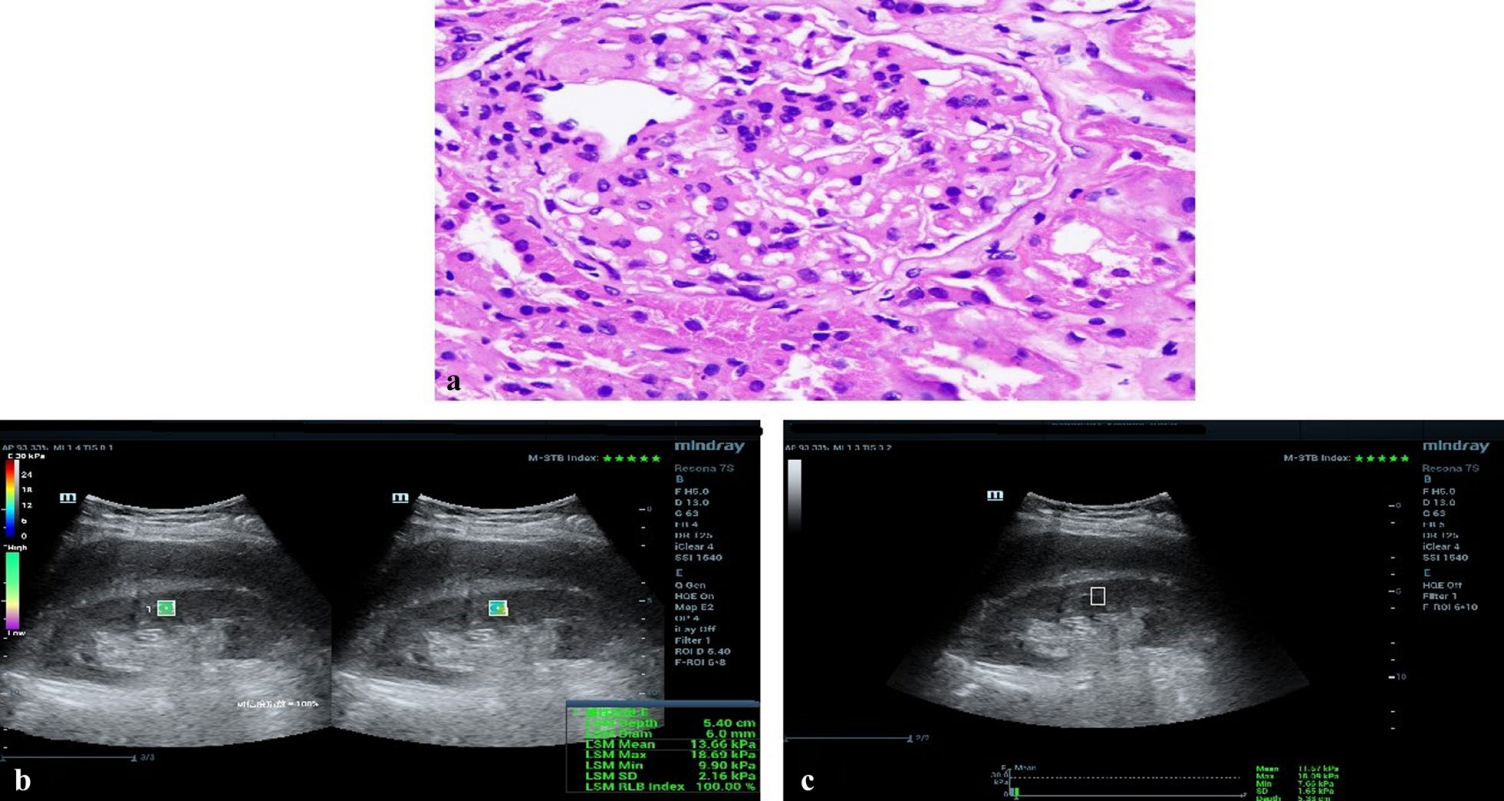
A 58-year-old man underwent renal biopsy and shear wave elastography of his right kidney. **a** The pathological examination revealed IgA nephropathy (H&E staining); **b** STE examination tested his Emax (18.69 kPa) and Emean (13.66 kPa) values; **c** STQ examination tested his Emax (16.09 kPa) and Emean (11.67 kPa) values. *STE* sound touch elastography, *STQ* sound touch quantification, *Emax* maximum elastic value, *Emean* mean elastic value

Both conventional US and SWE were performed with the convex SC6-1U probe (3.5–5 MHz) on the Mindray ultrasound system (Resona 7s, Shenzhen, China). All sonographic procedures were performed by a senior sonographer who was unaware of group assignments. After emptying bladder participants were scanned in the lateral recumbent position. STE and STQ measurements were obtained in a single region of interest (8 × 6 mm for STE, 6 × 10 mm for STQ), the middle area of right renal parenchyma with specific avoidance of renal pyramids. Three readings were taken per participant and maximum, and mean elastic values (Emax, Emean) were recorded as Young’s modulus in kPa (Fig. 2).

### Kidney biopsy

The percutaneous biopsy was performed under real-time ultrasound guidance, aimed to the lower pole of the right kidney (Fig. 2). The severity of renal impairment was categorized as mild (≤ 9), moderate (10–18), or severe (≥ 19) according to a previously published pathology scoring system [22].

### Statistical analysis

Categorical variables were expressed as numbers and percentages. The K–S test was performed to test the normality distribution for continuous data. For normally distributed data, variables were summarized as means (standard deviation, SD); while non-normally distributed, median and (interquartile range, IQR) was reported. Student's *t* test or One-Way ANOVA was used to determine whether or not there is a statistically significant difference between the means of two or more groups. Mann–Whitney *U* test or Wilcoxon rank-sum test was used to compare the differences between two or more groups if variables are not normally distributed. Pearson correlation analysis was used to analyze the relationship between two normally distributed variables, while Spearman's correlation was performed on two non-normally distributed variables. The receiver operating characteristic (ROC) curve, and area under the ROC curve (AUC) were calculated to determine the cutoff values for differentiating normal and renal pathologies. Statistical analyses were conducted using SPSS version 26.0 (SPSS Inc, Chicago, IL, USA), setting the statistical significance as a *p* value < 0.05.

## Results

### Patients’ characteristics

In this study, there were 60 cases, 37 were male and 23 were female. The mean age was 40.85 ± 15.06 years (range, 8–75 years). Forty-five healthy subjects were included as controls (10 males and 35 females). The mean age was 39.29 ± 15.36 years (range, 23–72 years). Mean height, weight and BMI were 1.68 ± 0.09 m, 71.50 ± 15.00 kg, 25.21 ± 3.99 kg/m^2^ for cases, and 1.65 ± 0.06 m, 65.93 ± 11.20 kg, 24. 15 ± 3.46 kg/m^2^ for controls. There were no significant differences between cases and controls in terms of age, height or BMI. The body weight was significantly higher for cases than controls (*p* < 0.05) (Table 1).

**Table 1:** Demographics of patients with proteinuria and control subjects

	Control group (*n* = 45)	Case group (*n* = 60)	*T* value	*p* value
Gender			–	0.000**
Female, *n* (%)	35 (77.78)	23 (38.33)		
Male, *n* (%)	10 (22.22)	37 (61.67)		
Age (year)	39.29 ± 15.36	40.85 ± 15.06	− 0.521	0.603
Height (m)	1.65 ± 0.06	1.68 ± 0.09	− 1.705	0.091
Weight (kg)	65.93 ± 11.20	71.50 ± 15.00	− 2.089	0.039*
BMI (kg/m^2^)	24.15 ± 3.46	25.21 ± 3.99	− 1.431	0.156

*BMI* body mass index

*p<0.05; **p<0.01

Kidney biopsy findings revealed membranous nephropathy in 19 cases, IgA nephropathy in ten, minimal change nephropathy in eight, allergic interstitial nephritis in six, allergic purpura nephritis in six, focal segmental glomerulosclerosis in three, systemic lupus erythematosus with nephritis in two and other pathological types in six.

### Maximum and minimum elastic modulus

STE and STQ findings of right kidney were listed in Table 2. It was demonstrated that Emax and Emean were significantly different between cases and controls for STE, but not for STQ.

**Table 2:** Comparison of Emax and Emean values measured by STE and STQ between cases and controls

	Control group (*n* = 45)	Case group (*n* = 60)	*T* value	*p*
STE (kPa)				
Emean	9.83 ± 3.21	12. 14 ± 3.84	− 3.271	0.001**
Emax	14.70 ± 4.61	17.81 ± 5.64	− 3.020	0.003**
STQ (kPa)				
Emean	10.65 ± 2.86	11.47 ± 3.36	− 1.303	0.196
Emax	17.19 ± 4.98	19.10 ± 5.75	− 1.784	0.077

*STE* sound touch elastography, *STQ* sound touch quantification, *Emax* maximum elastic value, *Emean* mean elastic value

*p<0.05; **p<0.01

### ROC curve analysis

**Fig. 3:**
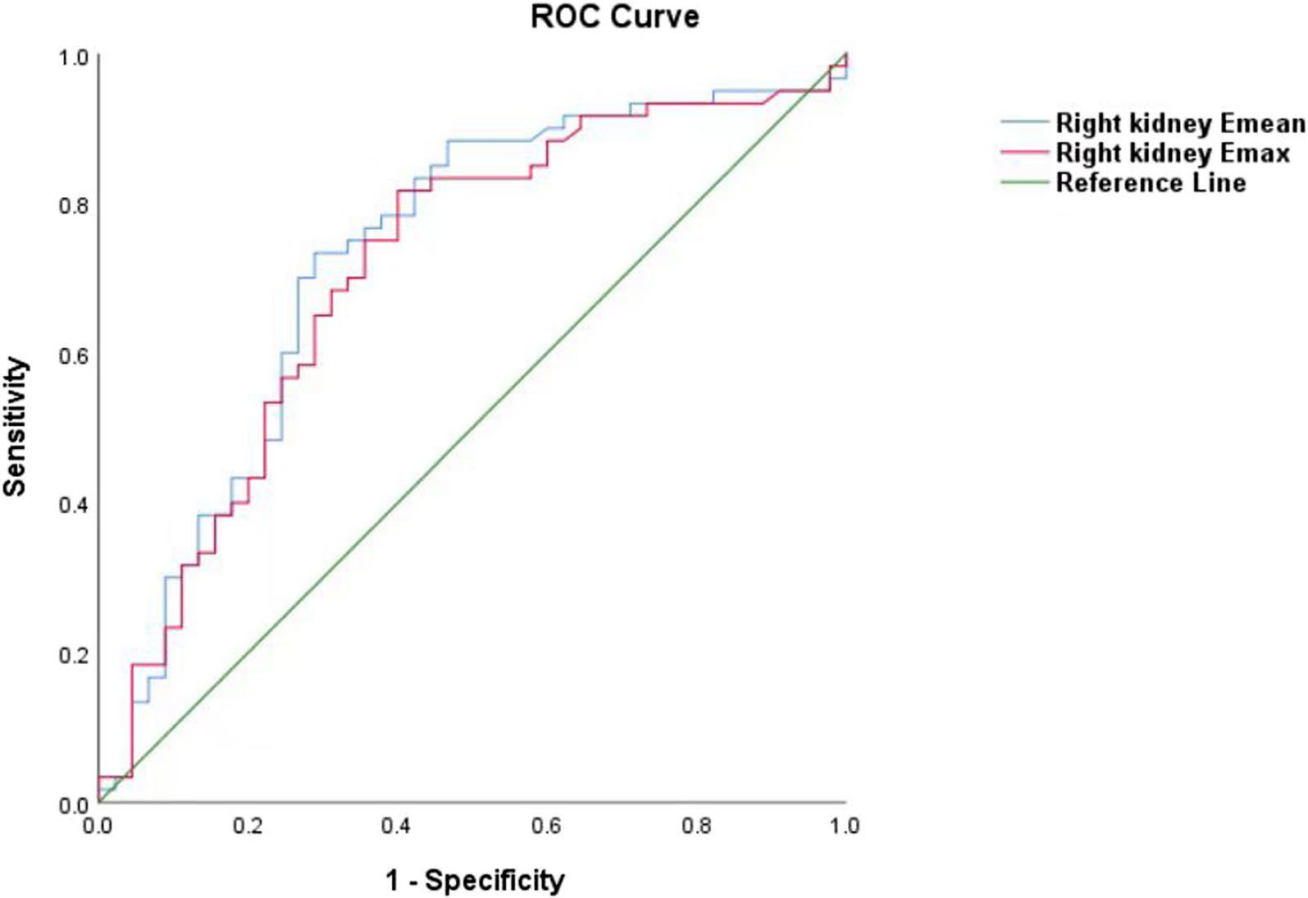
ROC curve of STE measurements for Emax (AUC= 0.718) and Emean (AUC= 0.744) values to diagnose nephropathy. *STE* sound touch elastography, *ROC* receiver operating characteristic, *Emax* maximum elastic value, *Emean* mean elastic value

ROC analysis of STE measurements revealed that using a cutoff of 13.53 kPa for Emax, the AUC to distinguish nephropathy from healthy was 0.718 (95% confidence intervel [CI]: 0.615–0.821), with a sensitivity and specificity of 82.76%, 59.09%, respectively (Table 3; Fig. 3). The Youden index was 0.418. While using a cutoff of 10.16 kPa for Emean, a value of 0.744 (95% CI: 0.643–0.846) for AUC diagnose nephropathy from healthy with a sensitivity and specificity of 75.86%, 70.45%, respectively (Table 3; Fig. 3). The Youden index was 0.463.

**Table 3:** The optimal diagnostic cut-off values of Emax and Emean measured by STE to diagnose nephropathy

	Cut-off value	Sensitivity (%)	Specificity (%)	Youden index
STE (kPa)				
Emean	10.16	75.86	70.45	0.463
Emax	13.53	82.76	59.09	0.418

*STE* sound touch elastography, *Emax* maximum elastic value, *Emean* mean elastic value

### Renal pathological features

The kidney biopsy findings showed that 24 patients had mild renal impairment, 27 had moderate and 9 severe. STE measurements of three renal impairment levels demonstrated significantly different Emean and Emax values for right kidney (Table 4); In addition, no significant difference of Emean or Emax values measured by STQ for right kidney was found among those different renal impairment levels.

**Table 4:** Comparison of Emax and Emean values measured by STE and STQ in patients with different pathological stages of nephropathy

Group	STE (kPa)		STQ (kPa)	
	Emean	Emax	Emean	Emax
Control group (*n* = 45)	9.83 ± 3.21	14.70 ± 4.61	10.65 ± 2.86	17.19 ± 4.98
Renal pathological stage (*n* = 60)				
Mild (*n* = 24)	11.65 ± 2.36	16.79 ± 3.74	10.84 ± 2.44	19.21 ± 4.76
Moderate (*n* = 27)	12.39 ± 3.35	18.47 ± 4.97	11.85 ± 3.64	18.88 ± 4.99
Severe (*n* = 9)	12.69 ± 7.43	18.58 ± 10.45	11.98 ± 4.55	19.45 ± 9.85
*F *value	3.779	3.535	1.077	1.071
*p*	0.013*	0.017*	0.362	0.365

### Relationship between SWE values and clinical parameters in proteinuria cases

**Fig. 4:**
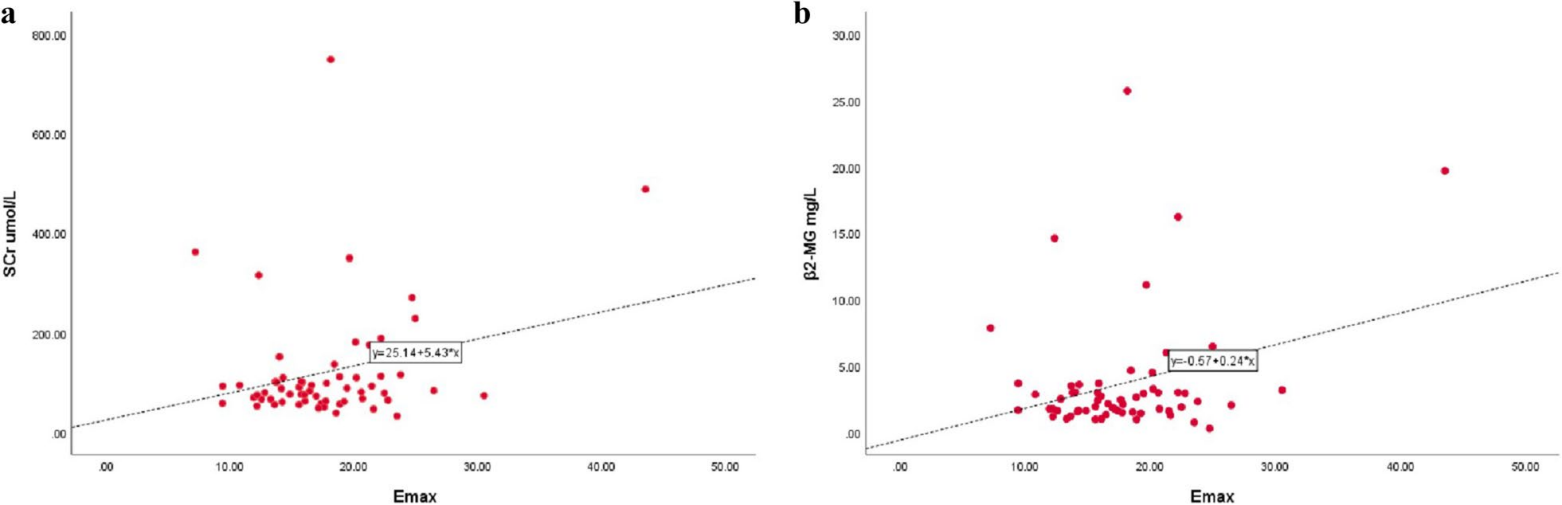
The Emax value for STE was positively related to Scr, β2-MG in the case group (*r* = 0.257, 0.292, *p* < 0.05). *STE* sound touch elastography, *Emax* maximum elastic value, *SCr* blood creatinine, *β2-MG* β2-microglobulin

There was a significantly positive correlation between Emax value for STE and Scr, β2-MG (*r* = 0.257, 0.292, *p* < 0.05) (Fig. 4). The correlation between Emax value for STE and eGFR (*r* = −0.135, *p* = 0.304) or 24-HUP (*r* = −0.05, *p* = 0.703) was negative, not significant. Furthermore, a positive insignificant correlation was observed between Emean values for STE and Scr, β2-MG (*r* = 0.128, 0.240, *p* = 0.328, 0.065), and a negative insignificant correlation between Emean for STE and eGFR, 24-HUP (*r* = −0.054, −0.117, *p* = 0.683, 0.374).

No correlations between Emean value for STQ and Scr, β2-MG, eGFR or 24-HUP. Meanwhile, there was no relationship between Emax values for STQ and Scr, β2-MG, eGFR or 24-HUP.

## Discussion and conclusions

In the present study, we found Emax and Emean measured by STE were significantly different between cases and controls. Additionally, both Emax and Emean values gradually increased as the renal impairment progressed from mild, moderate to severe. Our findings are consistent with another study which investigated 75 CKD patients undergoing renal biopsy and SWE [23]. It revealed Young’s modulus was associated with the patient's renal pathological scores, particularly with tubulointerstitial score (*ρ* = 0.442, *p* < 0.001) and glomerular score (*ρ* = 0.375, *p* = 0.001). The relationship between renal fibrosis and stiffness is still unclear. One study confirmed a positive relationship between renal fibrosis score and SWV [19], while another study showed no association existed [24]. It is likely the decline of renal blood perfusion played a stronger role than tissue fibrosis in renal stiffness [25]. Controversially, another study concluded that there was no relationship between SWV and renal fibrosis or blood perfusion [24].

Unlike the STE findings, Emax and Emean measured by STQ were not significantly different between cases and controls in this study. We speculated this discrepancy was due to different STE and STQ mechanisms. Compared with STE, STQ usually indicates the average rather than maximum elastic modulus of the ROI. Conversely, STE provides the maximum elastic modulus of ROI, displays a real-time colored stiffness map and reduces the influence of reverberation artifact [20, 21].Thus STE is superior to STQ in reflecting renal stiffness.

Elasticity can be measured from an ROI and can be displayed as the maximum (Emax), and mean (Emean) of Young’s modulus elasticity measurements. One study found that both Emax and Emean were effective to investigate breast lesions. Two other studies demonstrated that Emax was a better SWE parameter than Emean to distinguish benign from malignant breast lesions [26, 27]. To the best of our knowledge, this is the first study to compare STE performance with that of STQ for renal stiffness. We performed STE measurements and found Emax had a higher sensitivity than Emean (STE: 82.76 vs. 75.86%). However, the Youden index was low (STE: 0.418). The Emax cut-off value (STE: 13.53 kPa) suggested a considerable diagnostic accuracy and high false negative rate.

In this study, the association between elastic and clinical parameters was complicated, making it difficult to draw a definitive conclusion. Similarly, both positive and negative relationship between SWV and eGFR was found in various papers [28, 14]. It is reasonable to assume that renal stiffness is not dramatically correlated with Scr, β2-MG, eGFR, or 24hUP, which probably results from diverse pathological changes. Thus it’s essential to subgroup pathological types in further research.

There are some limitations to this study. Breathing and excess belly fat might affect the reliability of SWE results; we didn’t analyze the impact of age, blood perfusion and pressure or cardiovascular diseases on renal stiffness; the sample size is small. It is necessary to compare different pathological types in a larger population in future work.

In conclusion, both STE and STQ are promising, non-invasive, feasible methods to quantitatively evaluate renal stiffness for CKD patients with proteinuria. STE is more effective than STQ to assess renal stiffness.

## Data Availability

The datasets used and/or analyzed during the current study are available from the corresponding author on reasonable request.

## Abbreviations

ARFI: Acoustic radiation force impulse
AUC: Area under the receiver operating characteristic curve
BMI: Body mass index
CI: Confidence interval
CKD: Chronic kidney disease
eGFR: Estimated glomerular filtration rate
Emax: Maximum elastic modulus
Emean: Mean elastic modulus
24-HUP: 24-Hour urine protein
IQR: Interquartile range
ROC: Receiver operating characteristic curves
ROI: Region of interest
SCr: Serum creatinine
SD: Standard deviation
STE: Sound touch elastography
STQ: Sound touch quantification
SWE: Shear wave elastography
SWV: Shear wave velocity
US: Ultrasonograhy
β2-MG: β2-Microglobulin
